# Prognostic significance of the pretreatment controlling nutritional status score in colorectal cancer patients: an updated meta-analysis with 24 cohort studies

**DOI:** 10.3389/fnut.2025.1560355

**Published:** 2025-05-30

**Authors:** Jingkun Shang, Jinlai Wei

**Affiliations:** ^1^Department of Gastrointestinal Cancer Center, Chongqing University Cancer Hospital, Chongqing, China; ^2^Department of Gastrointestinal Surgery, The First Affiliated Hospital of Chongqing Medical University, Chongqing, China

**Keywords:** colorectal cancer, controlling nutritional status score, overall survival, recurrence-free survival, disease-free survival, cancer-specific survival, progression-free survival, meta-analysis

## Abstract

**Background:**

The prognostic significance of the Controlling Nutritional Status (CONUT) score in colorectal cancer has been extensively reported, yet it remains unclear. This study aims to conduct an updated meta-analysis to evaluate the association between pretreatment CONUT score and long-term oncological outcomes in patients diagnosed with colorectal cancer.

**Methods:**

A comprehensive literature search was conducted in PubMed, Embase, and Web of Science to identify eligible studies from inception to September 01, 2024, with an update on December 23, 2024. The primary endpoints evaluated were survival outcomes. Hazard ratios (HRs) with corresponding 95% confidence intervals (CIs) for survival outcomes were either extracted or calculated. A random-effects model was applied to pool all of the results. Statistical analyses were performed using R software, version 4.2.1.

**Results:**

A total of 24 retrospective cohort studies including 9,628 colorectal cancer patients were included. The pooled results demonstrated that patients with higher CONUT score exhibited significantly poorer outcomes across multiple survival metrics: overall survival (HR = 1.73; 95%CI: 1.50–2.01; *P* < 0.01; *I*^2^ = 62%), recurrence-free survival (HR = 1.51; 95% CI: 1.22–1.87; *P* < 0.01; *I*^2^ = 14%), disease-free survival (HR = 1.61; 95% CI: 1.33–1.95; *P* < 0.01; *I*^2^ = 35%), and cancer-specific survival (HR = 3.94; 95% CI: 2.34–6.62; *P* < 0.01; *I*^2^ = 0%). Furthermore, an additional study indicated that the pre-treatment CONUT score may serve as a potential prognostic indicator for progression-free survival in colorectal cancer patients undergoing first-line chemotherapy (*P* < 0.05).

**Conclusion:**

Our study demonstrates that the pretreatment CONUT score can serve as a valuable biomarker for predicting long-term oncological outcomes in patients with colorectal cancer.

## 1 Background

Colorectal cancer (CRC) remains the third most frequently diagnosed malignancy and the second leading cause of cancer-related mortality globally (1). Despite significant advancements in surgery, chemotherapy, radiotherapy, targeted therapy, and immunotherapy for CRC patients, clinical outcomes remain suboptimal (2). Currently, the tumor-node-metastasis (TNM) classification system is widely recognized as the predominant method for stratifying CRC prognoses. However, it is well-documented that significant variability exists in patient outcomes within the same TNM stage, particularly in stages II and III (3). This heterogeneity indicates that TNM staging alone may not comprehensively capture the full spectrum of prognostic outcomes. Consequently, there is an urgent need to develop biomarkers that can enhance the accuracy of patient stratification and identify individuals with adverse prognoses.

There is mounting evidence indicating that the nutritional status of cancer patients significantly influences both short-term treatment outcomes and long-term survival (4). Consequently, multiple nutritional indicators, including the NRS 2002 (5) and PG-SGA (6), have been utilized to predict the clinical outcomes of cancer patients. Nevertheless, those conventional nutritional assessment tools remain a subject of debate owing to their inherent complexity and susceptibility to subjective interpretation (4). Therefore, nutritional indicators derived from peripheral blood parameters have gradually emerged and continue to garner significant attention from researchers owing to their accessibility, non-invasiveness, and objectivity. Among them, the Controlled Nutritional Status (CONUT) score, which is calculated based on peripheral albumin levels, total cholesterol levels, and total lymphocyte counts, has emerged as a valuable nutritional screening tool (Table 1) (7). Recent literature extensively reports the clinical utility of the CONUT score in predicting both short- and long-term prognoses for solid tumors and hematologic malignancies (8). In 2015, Iski et al. (9) were the first to report the impact of the CONUT score on the prognosis of CRC patients undergoing radical surgery. Subsequently, numerous studies have further investigated the relationship between the CONUT score and clinical outcomes in CRC patients (10–12). In 2020, Takagi et al. (13) conducted a meta-analysis of six studies, preliminarily confirming the prognostic value of CONUT score in patients with CRC. However, they acknowledged that the number of included studies was limited, leaving the prognostic role of the CONUT score in CRC patients somewhat inconclusive. Given the growing body of recent research, we performed an updated meta-analysis to further elucidate the association between the pretreatment CONUT score and long-term oncological outcomes in CRC patients.

**Table 1:** The scoring criteria for the Controlling Nutritional Status (CONUT) score.

**Parameters**	**Degree**			
	**Normal**	**Mild**	**Moderate**	**Severe**
**Albumin level (g/dl)**	≥3.50	3.00–3.49	2.50–2.99	<2.50
Score	0	2	4	6
**Cholesterol level (mg/dl)**	≥1,600	1,200–1,599	800–1,199	<800
Score	0	1	2	3
**Total lymphocyte count (/ml)**	≥180	140–179	100–139	<100
Score	0	1	2	3
**CONUT score**	0–1	2–4	5–8	9–12

## 2 Methods

### 2.1 Search strategy

The present meta-analysis adhered to the Preferred Reporting Items for Systematic Reviews and Meta-Analyses (PRISMA) guidelines (14). A comprehensive search of relevant studies was conducted in PubMed, Embase, and Web of Science from inception to September 01, 2024, with an update on December 23, 2024. The search strategy employed a combination of keywords: (CONUT) AND (((colorectal) OR (colon) OR (rectum) OR (rectal)) AND ((cancer) OR (tumor) OR (carcinoma))). The detailed search strategy for each database was presented in Supplementary Table S1. No language restrictions were applied during the search. Furthermore, the reference lists of included studies were meticulously reviewed for additional relevant reports. Two investigators (SJK and WJL) independently performed the search.

### 2.2 Study selection

The inclusion criteria were as follows: (1) Studies that examined the association between the pretreatment CONUT score and survival outcomes in patients with CRC, including overall survival (OS), recurrence-free survival (RFS), disease-free survival (DFS), cancer-specific survival (CSS) and progression-free survival (PFS); (2) Hazard ratios (HRs) along with 95% confidence intervals (CIs) were either directly reported or could be calculated from the original literature; (3) The specific cut-off value for the CONUT score was clearly defined. The exclusion criteria were as follows: (1) Studies that did not provide separate data for CRC patients; (2) Case reports, reviews, conference papers, and letters; (3) Duplicate or overlapping datasets.

### 2.3 Data extraction and quality assessment

Two independent reviewers (SJK and WJL) conducted data extraction and cross-verified all results. The extracted data included critical information such as the first author, publication year, study period, country, study design, sample size, cut-off value of the CONUT score, and clinicopathological features including age, sex, primary treatment, tumor stage, tumor location, as well as survival outcomes and follow-up duration. The quality of the included studies was rigorously assessed using the Newcastle-Ottawa Scale (NOS) (15), which comprises eight predefined items. Each study was assigned a final score ranging from 0 to 9 based on a thorough evaluation; scores of 7–9 were deemed indicative of high-quality research.

### 2.4 Statistical analysis

The HRs along with their corresponding 95% CIs were used as the effect size for survival outcomes. When survival data were not directly reported in the literature, we extracted them from the survival curves using the methods outlined by Tierney et al. (16). Statistical heterogeneity among the included studies was evaluated using I^2^ statistics, and an *I*^2^ value of ≥ 50% was considered indicative of significant statistical heterogeneity. A random-effects model was utilized to synthesize HRs during the meta-analysis, given the substantial heterogeneity in clinical backgrounds across studies. Subgroup, sensitivity, and meta-regression analyses would be conducted to identify the potential sources of heterogeneity and evaluate the robustness of the pooled results in the presence of significant heterogeneity. A funnel plot along with Begg's and Egger's tests, was utilized to evaluate potential publication bias. For pooled outcomes exhibiting significant publication bias (Begg's test or Egger's test *P* < 0.1), the trim-and-fill method was further applied. A two-tailed *P* < 0.05 for pooled outcomes was considered statistically significant. All statistical analyses were performed using R software, version 4.2.1.

## 3 Results

### 3.1 Study characteristics

**Figure 1:**
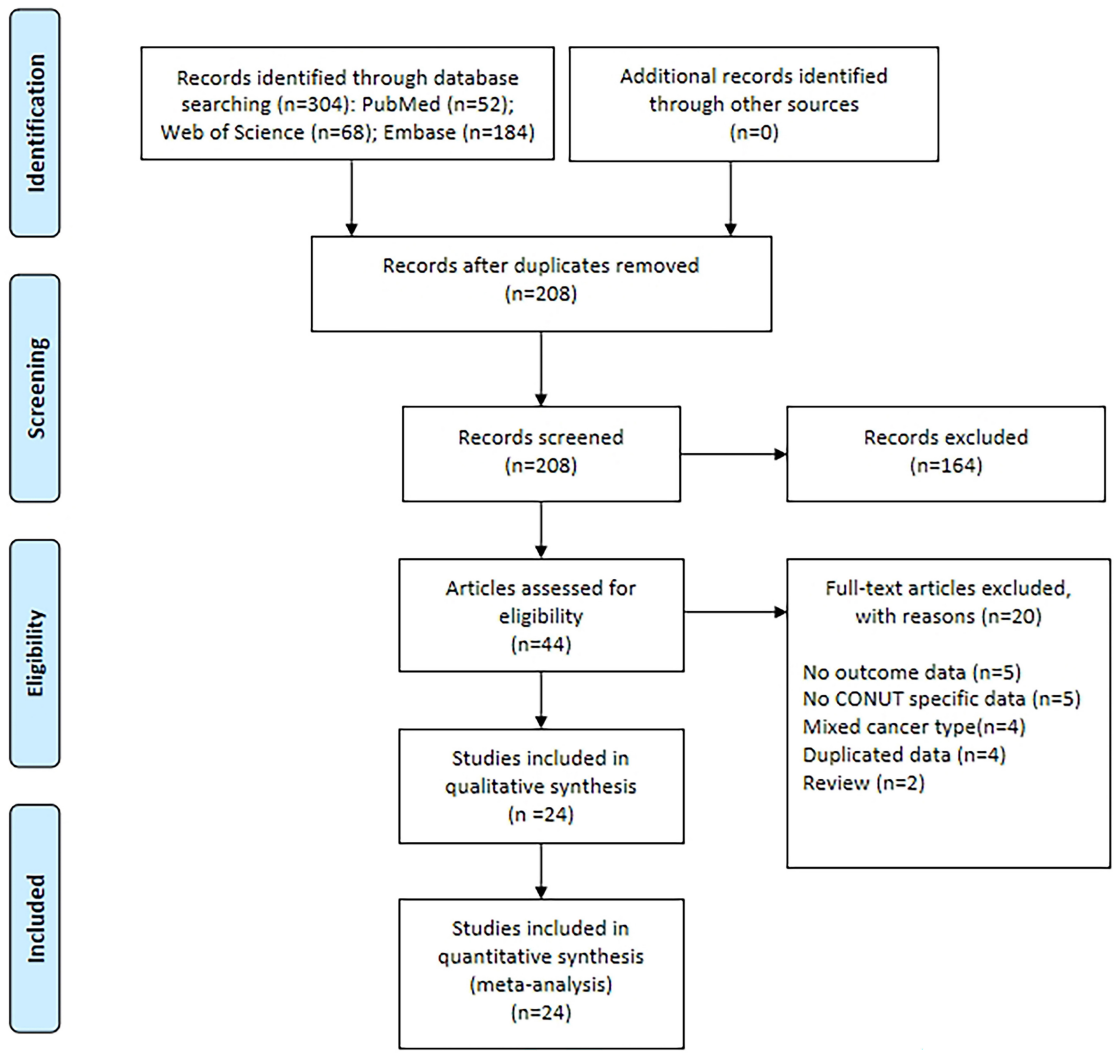
The PRISMA flowchart of study selection.

The database search yielded a total of 304 records, as shown in Figure 1. After a thorough evaluation of titles, abstracts, and full texts, 24 studies (9–12, 17–36) were ultimately included in this analysis. Tables 2, 3 provide comprehensive summaries of the basic characteristics and survival information of these included studies, respectively. Briefly, this meta-analysis encompassed a total of 9,628 patients from China, Japan, Korea, Turkey, Spain, and Italy. The publication years ranged from 2015 to 2024, with sample sizes varying between 57 and 1,112 individuals. Among the included studies, 19 focused on colorectal cancer, 4 on colon cancer, and 1 on rectal cancer. Regarding primary treatment modalities, 20 studies involved surgery, 2 studies involved mixed treatments, while neoadjuvant chemoradiotherapy and first-line chemotherapy were employed in 1 study each. Nineteen studies evaluated OS, 8 assessed RFS, 8 evaluated DFS, 4 evaluated CSS, and 1 assessed PFS. Notably, these studies demonstrated good quality, with NOS scores ranging from 6 to 7 (Table 3, Supplementary Table S2).

**Table 2:** Basic information of included studies.

**Reference**	**Country**	**Study design**	**Study interval**	**Age, years (median/mean)**	**Sex (male/female)**	**Primary treatment**	**Tumor location**	**TNM stage**	**NOS**
Iseki et al. (9)	Japan	Retrospective	2004–2009	69.79	112/92	Surgery	Colorectal	II–III	7
Galizia et al. (10)	Italy	Retrospective	2004–2014	NA	334/228	Surgery	Colorectal	I–IV	7
Tokunaga et al. (11)	Japan	Retrospective	2005–2014	68 (range, 19–93)	247/170	Surgery	Colorectal	I–III	7
Daitoku et al. (12)	Japan	Retrospective	2005–2014	63.0 (range, 34–86)	126/85	First-line chemotherapy	Colorectal	IV	7
Yamamoto et al. (17)	Japan	Retrospective	2007–2015	NA	291/231	Surgery	Colorectal	I–IV	6
Yang et al. (18)	China	Retrospective	2015–2017	58.4 ± 11.8	90/70	Surgery	Colorectal	I–III	7
Hayama et al. (19)	Japan	Retrospective	2012–2017	67 (range, 22–93)	180/121	Surgery	Colorectal	I–III	7
Horie et al. (21)	Japan	Retrospective	2004–2013	≥75	241/183	Surgery	Colon	I–III	6
Sato et al. (25)	Japan	Retrospective	2013–2019	72.2 ± 11.8	34/23	Surgery	Colorectal	II–III	6
Takamizawa et al. (20)	Japan	Retrospective	2001–2015	61.0 (range, 20–91)	573/423	Mixed	Colorectal	IV	7
Xie et al. (24)	China	Retrospective	2012–2014	58.48 ± 13.22	324/188	Surgery	Colorectal	I–III	7
Akabane et al. (22)	Japan	Retrospective	2008–2018	66 (range, 33–96)	239/160	Surgery	Colorectal	IV	7
Hiramatsu et al. (23)	Japan	Retrospective	2008–2018	65 (range, 27–98)	461/360	Surgery	Colorectal	III	7
Güç et al. (26)	Turkey	Retrospective	2010–2014	59 (range, 19–87)	108/77	Mixed	Colorectal	IV	7
Jin et al. (27)	China	Retrospective	2012–2015	60.8 (range, 25–90)	259/217	Surgery	Colorectal	I	7
Martínez-Escribano et al. (28)	Spain	Retrospective	2011–2019	≥70	185/140	Surgery	Colorectal	II–III	6
Mazaki et al. (29)	Japan	Retrospective	2000–2015	69 (range, 30–91)	336/206	Surgery	Colon	II–III	7
Pian and Oh (30)	Korea	Retrospective	2010–2015	63 (range, 25–87)	183/122	Surgery	Colorectal	I	7
Xie et al. (31)	China	Retrospective	2012–2015	66 (range, 19–89)	66/60	Surgery	Colorectal	I–IV	6
Kim et al. (32)	Korea	Retrospective	2004–2014	NA	667/445	Surgery	Colon	I–III	7
Lu et al. (33)	China	Retrospective	2012–2022	61 (IQR, 54–68)	205/95	Neoadjuvant chemoradiotherapy	Rectal	0–III	6
Okamoto et al. (34)	Japan	Retrospective	2006–2020	65 (IQR, 58–72)	109/36	Surgery	Colorectal	IV	7
Cozzani et al. (35)	Italy	Retrospective	2013–2018	72.2 (range, 38–95)	172/169	Surgery	Colon	I–IV	7
Liu et al. (36)	China	Retrospective	2015–2019	58.4 ± 12.7	92/125	Surgery	Colorectal	I–III	7

IQR, Inter-quartile range; NOS, Newcastle-Ottawa Scale.

**Table 3:** Survival information of included studies.

**Reference**	**Sample size**	**Cut-off value**	**Low CONUT group**	**High CONUT group**	**Median follow-up time, months**	**Survival outcomes**	**Multivariate analysis**	**Confounders adjusted**
Iseki et al. (9)	204	0–2/≥3	150	54	NA	CSS; RFS	Yes/Yes	1, 2, 3, 4, 5, 7, 8, 11, 12
Galizia et al. (10)	562	0–2/≥3	418	144	34.7 (IQR, 14.3–70.5)	OS; DFS	Yes/Yes	3, 4, 5, 6, 10, 11, 12
Tokunaga et al. (11)	417	0–1/2–4/>4	Normal:246; Light:127; Moderate/Severe:44		38.0 (range, 1–115)	OS; RFS	Yes/Yes	1,2,3,5,12
Daitoku et al. (12)	211	PFS:0–1/2–4/>4 OS:0–4/>4	Normal:89; Light:90; Moderate/Severe:32		NA	OS; PFS	Yes/No	1,2,3,5,12
Yamamoto et al. (17)	552	0–2/≥3	364	158	NA	OS	No	-
Yang et al. (18)	160	0–2/≥3	86	74	30 (range, 6–42)	CSS; RFS	Yes/Yes	1, 2, 3, 4, 5, 6, 7, 8, 11, 12
Hayama et al. (19)	301	0–2/≥3	106	195	46.4 (range, 0.7–78.4)	OS; RFS	Yes/Yes	1, 2, 3, 5, 6, 7, 8, 12
Horie et al. (24)	424	0–1/2–4/>4	Normal:261; Light:148; Moderate/Severe:15		61.3 (range, 0.3–147.3)	OS	No	-
Sato et al. (25)	57	0–6/≥7	44	13	26	CSS; DFS	Yes/Yes	1,2,3,5,7,8, 11,12
Takamizawa et al. (20)	996	0–1/2–3/≥4	Normal:614; Light:276; Moderate/Severe:106		53 (range, 1–228)	OS	Yes	1,2,3,5,6,8,12
Xie et al. (21)	512	0–1.5/>1.5	246	266	64 (range, 1–80)	OS; DFS	Yes/Yes	1,2,3,4,5,7,8,9,12
Akabane et al. (22)	337	0–1/2–4/5–8/9–12	Normal:140; Light:130; Moderate:59; Severe:8		NA	OS	Yes	1,2,5,6,7, 12
Hiramatsu et al. (23)	821	0–1/≥ 2	455	366	53.0 (range, 1–119)	OS; RFS	Yes^#^/Yes^#^	1,3,7,11,12
Güç et al. (26)	185	0–4/≥ 5	69	116	38.4 (range, 2–120)	OS	Yes	1,2,5,12
Jin et al. (27)	476	0–2/≥ 3	NA	NA	68 (range, 4–84)	OS; DFS	Yes/No	1,2,3,5,12
Martínez-Escribano et al. (28)	325	0–4/≥ 5	227	98	NA	OS	Yes	1,2,12
Mazaki et al. (29)	542	0–1/≥ 2	NA	NA	73.2 (range, 0.2–225.2)	RFS	No	-
Pian and Oh (30)	305	0–2/≥ 3	NA	NA	87.0 (range,3–125)	OS; DFS	Yes/Yes	1,2,3,5,6,7,8,9,12
Xie et al. (31)	126	0–1/2–4/>4	Normal:57; Light:56; Moderate/Severe:13		72 (range: 2–101)	OS; RFS	No/No	-
Kim et al. (32)	1,112	0–1/2–4/>4	Normal:649; Light:397; Moderate/Severe:66		NA	OS	Yes	1, 2, 3, 4, 7, 8, 12
Lu et al. (33)	300	0–4/≥5	259	41	NA	OS; DFS	Yes/No	1, 2, 3, 4, 5, 7, 8, 9, 10, 12
Okamoto et al. (34)	145	0–3/≥4	130	15	NA	OS; DFS	Yes/No	4, 5, 7, 12
Cozzani et al. (35)	341	0–2/≥3	204	97	≥ 60	OS; DFS	No/No	-
Liu et al. (36)	217	0–4/≥5	189	28	49.6 (range, 8–85)	CSS; RFS	Yes/Yes	1, 2, 3, 5, 12

CONUT, the Controlling Nutritional Status score; OS, Overall survival; RFS, Recurrence-free survival; DFS, Disease-free survival; PFS, Progression-free survival; CSS, Cancer-specific survival; IQR, Inter-quartile range; NA, Not available.

# That the inverse probability of treatment weighting (IPTW) analysis was performed by Hiramatsu et al. (23) is considered as a multivariate analysis in the present study.

Confounders adjusted by multivariate analysis included two categories: (1) common covariates (1: age; 2: sex; 3: tumor markers; 4: tumor size; 5: tumor location; 6: tumor differentiation; 7: TNM stage; 8: lymphatic vascular invasion; 9: peripheral nerve invasion; 10: tumor deposit; 11: adjuvant chemotherapy), and (2) additional covariate (12: others like BMI and other biomarkers). All the included studies that evaluated multivariate analyses of two survival indicators were analyzed using the same covariates.

### 3.2 Relationship between the CONUT score and OS

**Figure 2:**
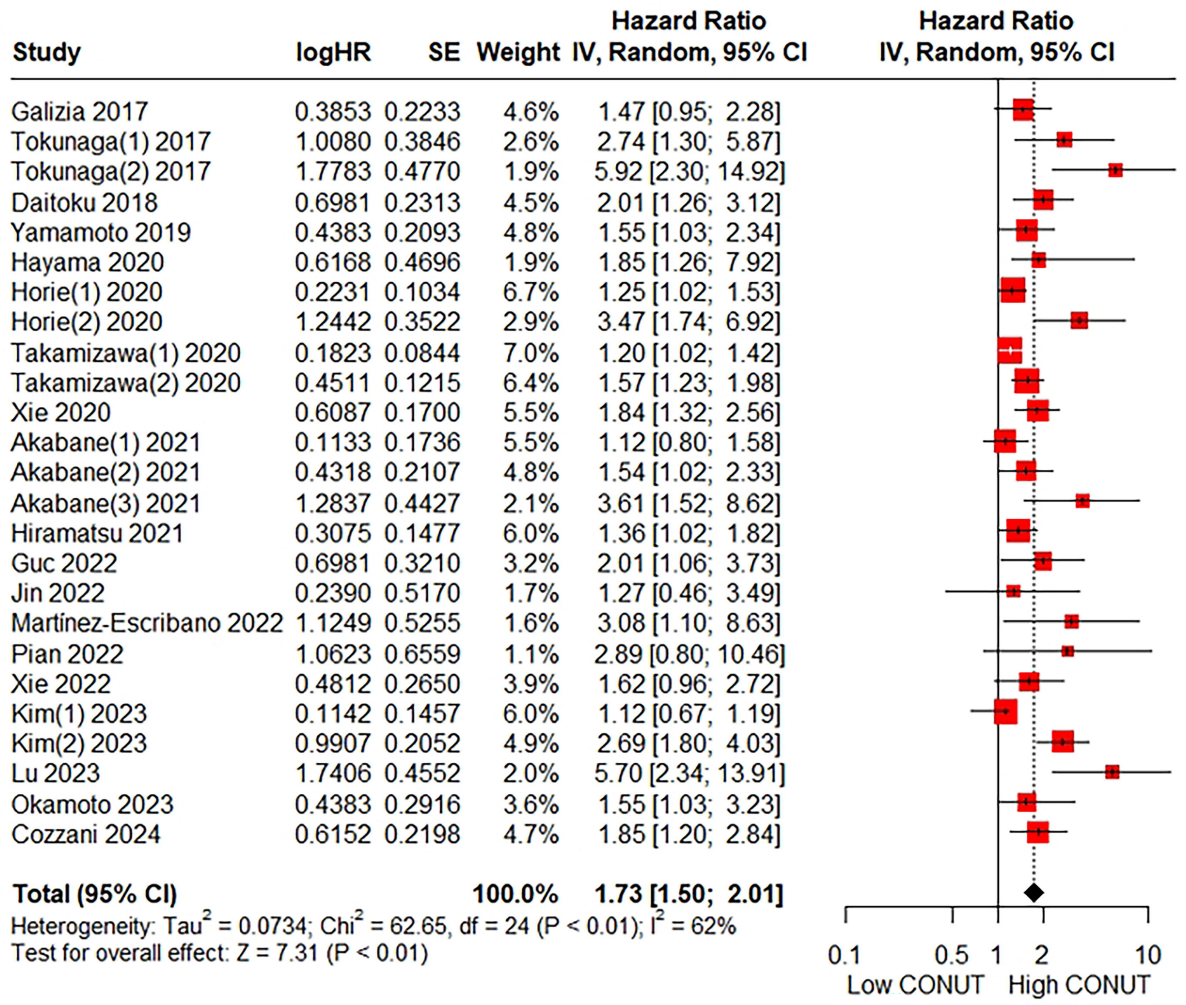
Forest plot assessing the relationship between the CONUT score and OS.

The association between the CONUT score and OS was examined in 19 studies encompassing 8,510 patients. The pooled HR was 1.73 (95% CI: 1.50–2.01; *P* < 0.01), indicating a significant correlation between a higher CONUT score and poorer OS in CRC patients (Figure 2). Given the substantial heterogeneity observed (*I*^2^ = 62%), subgroup analyses were conducted to explore the stability of the pooled result across various factors, including publication year (< 2020 vs. ≥2020), country (China vs. Japan vs. Others), sample size (< 300 vs. ≥300), primary treatment modality (Surgery vs. Others), reference value for CONUT score (Normal vs. Others), TNM stage (Non-metastatic vs. Mixed vs. Metastatic), tumor location (Colorectal cancer vs. Colon cancer vs. Rectal cancer), multivariate analysis (≥5 common covariates adjusted vs. < 5 common covariates adjusted vs. Univariate), and NOS (6 vs. 7). As presented in Table 4 and Supplementary Figure S1, all subgroup analyses consistently demonstrated that patients with a higher CONUT score had significantly reduced OS compared to those with a lower CONUT score. Additionally, a multivariate meta-regression analysis based on the above parameters was performed. As shown in Supplementary Table S3, none of these factors were found to be the origin of potential sources of heterogeneity. Furthermore, sensitivity analysis by sequentially omitting each study showed no significant alteration in the overall outcome (Supplementary Figure S2).

**Table 4:** Results of subgroup analyses of overall survival.

**Variables**	**Subgroups**	**Cohorts, *n***	**Patients, *n***	**HR (95%CI)**	***I*^2^ (%)**
	Total	25	8,510	1.73 (1.50–2.01)	62
Publication year	<2020	5	1,742	2.05 (1.42–2.95)	55
	≥2020	20	6,768	1.67 (1.42–1.97)	62
Country	China	4	1,414	2.05 (1.25–3.39)	56
	Japan	14	4,266	1.64 (1.36–1.97)	62
	Others	7	2,830	1.82 (1.34–2.47)	62
Sample size	≥300	21	7,843	1.77 (1.48–2.11)	67
	<300	4	667	1.80 (1.38–2.34)	0
Primary treatment	Surgery	20	6,818	1.72 (1.46–2.04)	57
	Others	5	1,692	1.86 (1.25–2.77)	77
Reference value of CONUT	Normal	12	4,169	1.81 (1.56–2.10)	0
	Others	13	4,341	1.73 (1.34–2.23)	75
TNM stage	Nonmetastatic	13	4,993	2.11 (1.56–2.85)	73
	Mixed	4	1,581	1.62 (1.29–2.02)	0
	Metastatic	8	1,936	1.50 (1.25–1.81)	52
Tumor location	Colorectal cancer	20	7,319	1.66 (1.43–1.94)	57
	Colon cancer	4	891	1.74 (1.19–2.55)	69
	Rectal cancer	1	300	5.70 (2.34–13.91)	/
Multivariate analysis^#^	≥5 common covariates adjusted	12	4,487	1.69 (1.34–2.12)	70
	<5 common covariates adjusted	8	2,580	1.96 (1.47–2.63)	46
	Univariate	5	1,443	1.66 (1.25–2.19)	59
NOS	6	6	1,727	2.13 (1.36–3.35)	74
	7	19	6,783	1.66 (1.43–1.94)	58

CONUT, the Controlling Nutritional Status score.

# That the inverse probability of treatment weighting (IPTW) analysis was performed by Hiramatsu et al. (23) is considered as a multivariate analysis in the present study.

### 3.3 Relationship between the CONUT score and RFS

**Figure 3:**
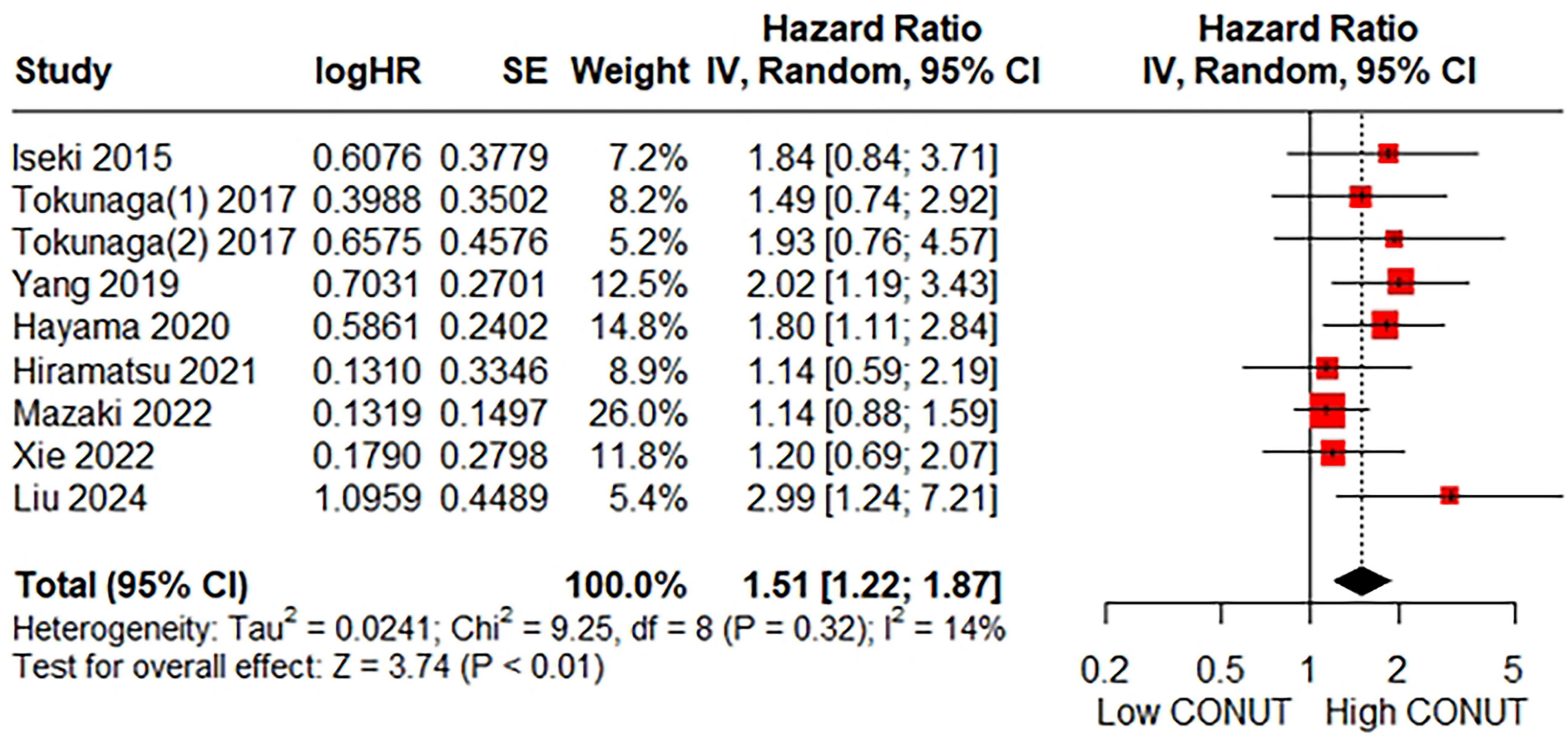
Forest plot accessing the relationship between the CONUT score and RFS.

A total of eight studies consisting of 2,788 patients reported on RFS. The pooled HR was HR = 1.51 (95%CI: 1.22–1.87; *P* < 0.01; *I*^2^ = 14%), indicating a significant association between a higher CONUT score and poorer RFS (Figure 3). Given the low heterogeneity of the pooled results, subgroup and sensitivity analyses were not performed.

### 3.4 Relationship between the CONUT and score DFS

**Figure 4:**
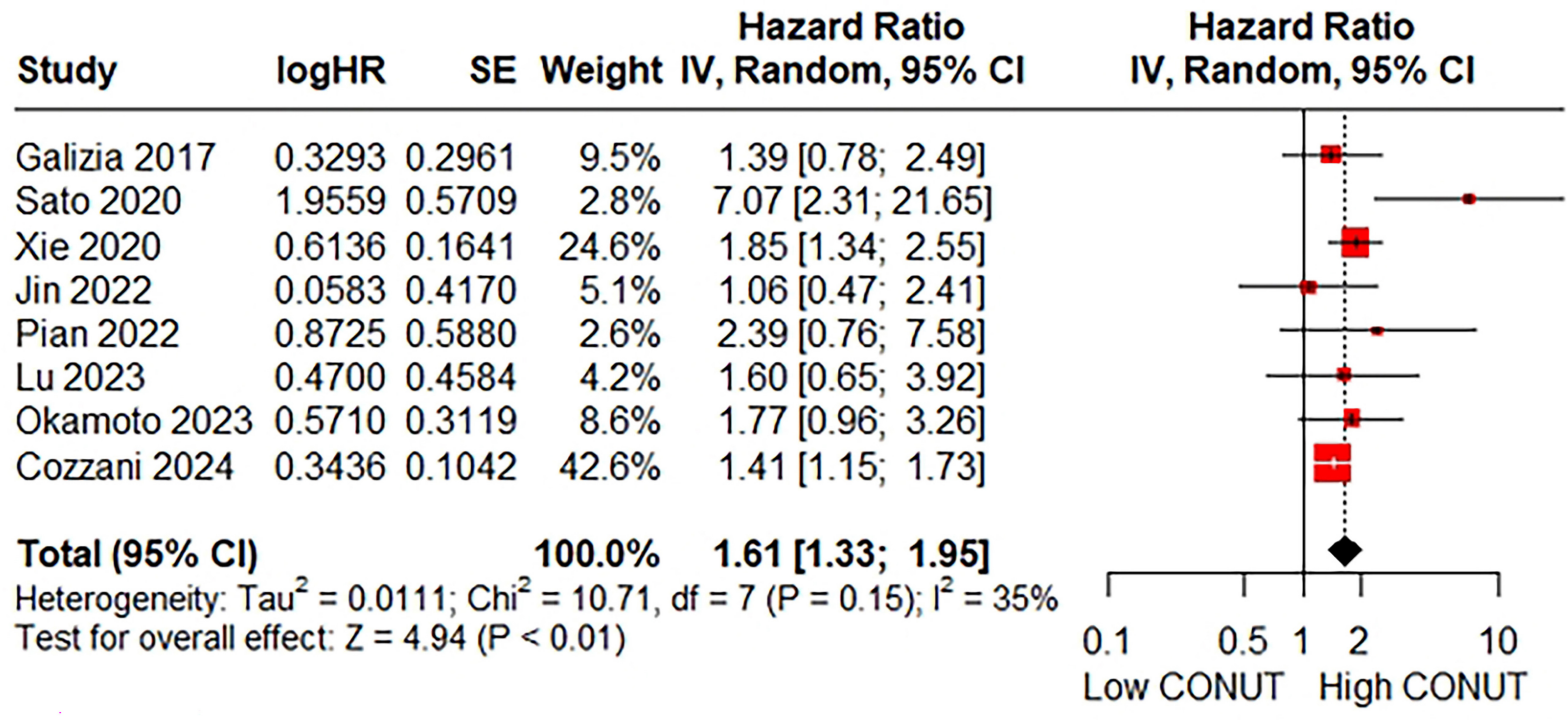
Forest plot accessing the relationship between the CONUT score and DFS.

The relationship between the CONUT score and DFS was assessed in eight studies involving 2,698 patients. The pooled HR was 1.61 (95% CI: 1.33–1.95; *P* < 0.01; *I*^2^ = 35%), suggesting a significant association between a higher CONUT score and poorer DFS (Figure 4). Similarly, due to the low heterogeneity of the pooled results, subgroup and sensitivity analyses were not conducted.

### 3.5 Relationship between the CONUT and score CSS

**Figure 5:**
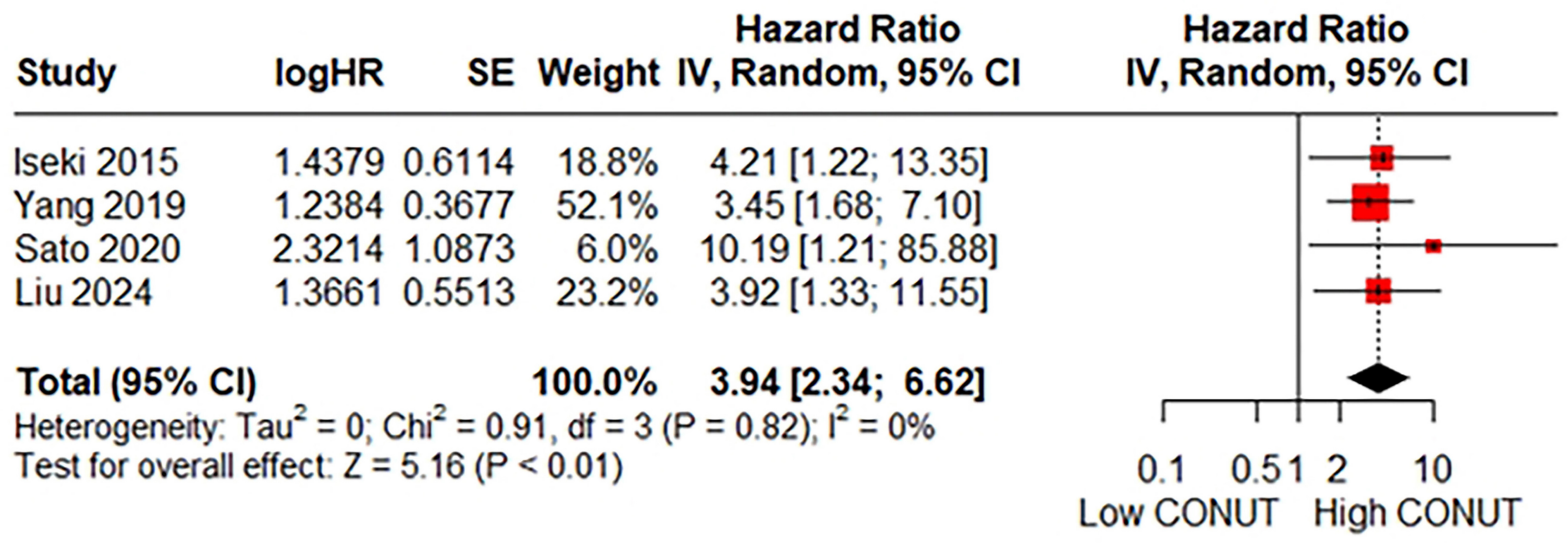
Forest plot accessing the relationship between the CONUT score and CSS.

A total of four studies consisting of 638 patients reported on CSS. The pooled HR was HR = 3.94 (95% CI: 2.34–6.62; *P* < 0.01; *I*^2^ = 0%), indicating a significant association between a higher CONUT score and poorer CSS (Figure 5). Due to the absence of heterogeneity, subgroup and sensitivity analyses were not performed.

### 3.6 Relationship between the CONUT and score PFS

Regarding the relationship between the CONUT score and PFS, only one study investigated this association in metastatic colorectal cancer patients receiving first-line chemotherapy. This study demonstrated that a high CONUT score was significantly associated with poor PFS (*P* < 0.05).

### 3.7 Publication bias

**Figure 6:**
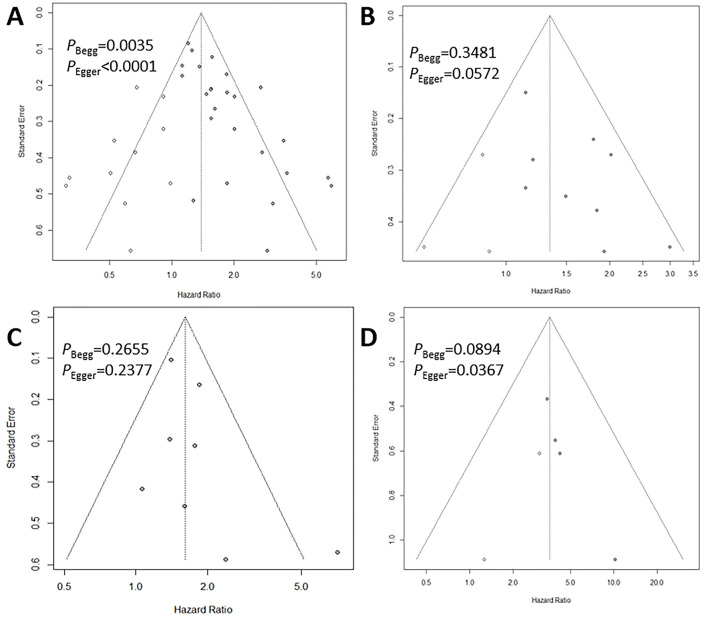
Begg's funnel plots assessing publication bias between the CONUT score and OS **(A)**, RFS **(B)**, DFS **(C)** and CSS **(D)**.

The funnel plots, combined with Begg's and Egger's tests for the survival outcomes, are presented in Figure 6. These analyses revealed significant publication bias for OS (Begg's *P* = 0.0035, Egger's *P* < 0.0001), RFS (Begg's *P* = 0.3481, Egger's *P* = 0.0572), and CSS (Begg's *P* = 0.0894, Egger's *P* = 0.0367). However, no significant publication bias was observed for DFS (Begg's *P* = 0.2655, Egger's *P* = 0.2377). Trim-and-fill analyses further demonstrated that the pooled results remained robust after accounting for hypothetical unpublished studies: 11 additional studies for OS (HR = 1.39, 95% CI: 1.14–1.69, *P* < 0.01, *I*^2^ = 71.8%), 3 for RFS (HR = 1.34, 95% CI: 1.09–1.66, *P* < 0.01, *I*^2^ = 35.2%), and 2 for CSS (HR = 3.59, 95% CI: 2.25–5.72, *P* < 0.01, *I*^2^ = 0%). Due to the inclusion of only one study, the publication bias test for PFS was not conducted.

## 4 Discussion

Malnutrition is highly prevalent among cancer patients, particularly in those with CRC, owing to factors such as malabsorption and bowel obstruction (37). Extensive evidence demonstrates that malnutrition can lead to prolonged hospital stays, increased postoperative complications, diminished sensitivity to postoperative treatments, and ultimately a poorer prognosis for cancer patients (38, 39). Therefore, the early identification and management of malnutrition are critical components of clinical practice for improving outcomes in CRC patients.

The CONUT score, as a nutritional assessment tool developed based on peripheral albumin levels, cholesterol levels, and total lymphocyte counts, possesses several advantages such as readily accessible data, straightforward calculation, non-invasive procedures, and objectivity (40). Consequently, an increasing number of studies have evaluated the prognostic value of CONUT score in various malignancies. In gastric cancer, a recent meta-analysis involving 9,764 patients demonstrated that patients in the high CONUT group exhibited poorer OS and RFS compared to those in the low CONUT group (4). Another meta-analysis by Liu et al. (41) confirmed that the CONUT score serves as a practical prognostic factor associated with the prognosis of biliary tract cancer. Additionally, the prognostic value of the CONUT score has been successfully validated in patients with head and neck cancer (42), breast cancer (43), hematological malignancies (44). In CRC, although a previous meta-analysis by Takagi et al. (13) in 2020 showed the significant efficacy of the CONUT score in predicting long-term survival, this study incorporated only 6 studies with 2,601 patients, resulting the conclusion unclear. Therefore, to further elucidate the prognostic value of CONUT score in CRC patients remains important.

By integrating data from 24 studies involving a total of 9,628 CRC patients, our meta-analysis revealed that patients in the high CONUT group had a 1.73-fold increased risk of poor OS. Given the substantial heterogeneity observed, subgroup analyses were conducted to investigate the robustness of the pooled results across various types of CRC patients. Consistently, the pretreatment CONUT score was identified as a significant prognostic biomarker across different regions, tumor locations, TNM stages, and primary treatments. However, despite sensitivity analysis supporting the stability of the findings, the sources of heterogeneity were not ascertained by meta-regression analysis. Additionally, it is important to acknowledge that the pooled result for OS showed significant publication bias. Nevertheless, after conducting trim-and-fill analysis, the pretreatment CONUT score remained a significant prognostic biomarker. Moreover, this meta-analysis demonstrated that patients in the high CONUT group faced a 1.51-, 1.61-, and 3.94-fold increased risk of poor RFS, DFS, and CSS, respectively. Notably, no significant heterogeneity was detected for these specific outcomes. Despite the presence of significant publication biases for RFS and CSS, the subsequent trim-and-fill analyses supported the reliability of these pooled results. Additionally, one included study initially examined the association between the CONUT score and PFS, yielding statistically significant results. Compared to the previous meta-analysis conducted 4 years ago (9), the present meta-analysis has several notable strengths. First, the larger sample size has narrowed the confidence intervals, enhancing the robustness of the findings. Second, our study encompassed a more diverse population with varying clinical characteristics, thereby increasing the generalizability of the prognostic value of the CONUT score. Finally, this study confirmed for the first time the predictive value of the CONUT score in DFS through meta-analysis.

The potential mechanism by which the CONUT score can effectively predict prognosis in CRC patients can be explained through the following aspects. First, serum albumin concentration serves as a critical indicator of liver function, nutritional status, and systemic inflammation (45). Pro-inflammatory cytokines such as IL-2 and IL-6 reduce albumin synthesis within hepatocytes, leading to decreased serum albumin levels (46). These cytokines also significantly promote cancer proliferation, invasion, and metastasis by accelerating cancer cell growth and compromising antitumor immunity via cytokine-mediated inflammatory responses (46). Furthermore, reduced serum albumin levels result in diminished synthesis of enzymes required for antibody production, weakened immune function, and compromised defense against tumors (47). Second, total cholesterol levels have been shown to strongly correlate with tumor growth and prognosis in various cancers (48). Although the precise role of cholesterol in cancer progression remains unclear, several studies have elucidated molecular mechanisms linking cancer progression and cholesterol metabolism (49, 50). Research suggests that an increased risk of cancer is inversely correlated with total serum cholesterol concentrations, possibly due to higher cholesterol content in tumor tissues compared to normal tissues, leading to reduced plasma cholesterol levels and caloric intake (49, 50). Additionally, mutations in genes involved in cholesterol metabolic pathways have been identified in cancer cells, potentially contributing to elevated intracellular cholesterol levels and promoting cancer cell growth (50). Finally, lymphocytes function as the primary effector cells of the immune system, orchestrating immune responses against tumor cells (51). Tumor-infiltrating lymphocytes produce a range of cytokines, including IFN-γ and TNF-α, which inhibit tumor growth and promote tumor cell apoptosis (52). CD8+ T cells can directly induce tumor cell death by releasing perforin and granzyme (53). Therefore, a decrease in lymphocyte count compromises the body's ability to effectively suppress tumor progression.

The current meta-analysis has several limitations. First, all included studies were retrospective in nature, which may introduce selection bias and highlights the need for further investigation through prospective studies. Second, the majority of the studies originated from Japan, indicating a potential regional bias and underscoring the necessity for more diverse international representation in future research. Finally, most patients underwent surgical treatment, limiting the generalizability of the CONUT score's predictive value in neoadjuvant therapy, first-line treatment, and subsequent lines of treatment, which require further exploration.

## 5 Conclusions

Our findings indicate that the pretreatment CONUT score may serve as a valuable prognostic biomarker for patients diagnosed with colorectal cancer, as individuals in the high CONUT group demonstrate significantly poorer long-term survival outcomes. Clinicians can leverage this informative indicator to stratify patients and tailor personalized treatment strategies. Nonetheless, additional research is warranted to validate the efficacy of this index in colorectal cancer prognosis.

## Data Availability

The original contributions presented in the study are included in the article/Supplementary material, further inquiries can be directed to the corresponding author.
