# Supplementary material for: Prognostic significance of the pretreatment controlling nutritional status score in colorectal cancer patients: an updated meta-analysis with 24 cohort studies
Source: Front Nutr. 2025 May 30;12:1560355. doi: 10.3389/fnut.2025.1560355 (PMC12162528; doi:10.3389/fnut.2025.1560355)
Supplement: Supplementary file 1 [file Data_Sheet_1.docx]

Table S1. Literature retrieval strategies and retrieval results.

| **Electronic database** | **Retrieval strategy** | **Retrieval result** |
| --- | --- | --- |
| PubMed | Search: **(CONUT) AND (((colorectal) OR (colon) OR (rectum) OR (rectal)) AND ((cancer) OR (tumor) OR (carcinoma)))** Sort by: Most Recent  "CONUT"[All Fields] AND (("colorectal"[All Fields] OR ("colon"[MeSH Terms] OR "colon"[All Fields] OR "colonic"[All Fields] OR "colons"[All Fields] OR "colon s"[All Fields] OR "colonal"[All Fields] OR "colonically"[All Fields] OR "colonitis"[All Fields]) OR ("rectum"[MeSH Terms] OR "rectum"[All Fields] OR "rectums"[All Fields]) OR ("administration, rectal"[MeSH Terms] OR ("administration"[All Fields] AND "rectal"[All Fields]) OR "rectal administration"[All Fields] OR "rectal"[All Fields])) AND ("cancer s"[All Fields] OR "cancerated"[All Fields] OR "canceration"[All Fields] OR "cancerization"[All Fields] OR "cancerized"[All Fields] OR "cancerous"[All Fields] OR "neoplasms"[MeSH Terms] OR "neoplasms"[All Fields] OR "cancer"[All Fields] OR "cancers"[All Fields] OR ("cysts"[MeSH Terms] OR "cysts"[All Fields] OR "cyst"[All Fields] OR "neurofibroma"[MeSH Terms] OR "neurofibroma"[All Fields] OR "neurofibromas"[All Fields] OR "tumor s"[All Fields] OR "tumoral"[All Fields] OR "tumorous"[All Fields] OR "tumour"[All Fields] OR "neoplasms"[MeSH Terms] OR "neoplasms"[All Fields] OR "tumor"[All Fields] OR "tumour s"[All Fields] OR "tumoural"[All Fields] OR "tumourous"[All Fields] OR "tumours"[All Fields] OR "tumors"[All Fields]) OR ("carcinoma"[MeSH Terms] OR "carcinoma"[All Fields] OR "carcinomas"[All Fields] OR "carcinoma s"[All Fields]))) | 52 |
| Embase | **(CONUT and ((colorectal or colon or rectum or rectal) and (cancer or tumor or carcinoma)))**.mp. [mp=title, book title, abstract, original title, name of substance word, subject heading word, floating sub-heading word, keyword heading word, organism supplementary concept word, protocol supplementary concept word, rare disease supplementary concept word, unique identifier, synonyms, population supplementary concept word, anatomy supplementary concept word] | 184 |
| Web of Science | **(CONUT) AND (((colorectal) OR (colon) OR (rectum) OR (rectal)) AND ((cancer) OR (tumor) OR (carcinoma)))** | 68 |

Table S2. Quality assessment of included studies using Newcastle-Ottawa Scale.

| **Reference** | **Representatives of the exposed cohort** | **Selection of the non-exposed cohort** | **Ascertainment of exposure** | **Was outcome of interest present at start of study** | **Comparability of cohorts on the basis of the design or analysis** | **Assessment of outcome** | **Was follow-up long enough for outcomes to occur** | **Adequate follow up** | **Total** |
| --- | --- | --- | --- | --- | --- | --- | --- | --- | --- |
| Iseki,2015 | 1 | 1 | 1 | 0 | 1 | 1 | 1 | 1 | 7 |
| Galizia,2017 | 1 | 1 | 1 | 0 | 1 | 1 | 1 | 1 | 7 |
| Tokunaga,2017 | 1 | 1 | 1 | 0 | 1 | 1 | 1 | 1 | 7 |
| Daitoku,2018 | 1 | 1 | 1 | 0 | 1 | 1 | 1 | 1 | 7 |
| Yamamoto,2019 | 1 | 1 | 1 | 0 | 1 | 0 | 1 | 1 | 6 |
| Yang,2019 | 1 | 1 | 1 | 0 | 1 | 1 | 1 | 1 | 7 |
| Hayama,2020 | 1 | 1 | 1 | 0 | 1 | 1 | 1 | 1 | 7 |
| Horie,2020 | 1 | 1 | 1 | 0 | 1 | 0 | 1 | 1 | 6 |
| Sato,2020 | 1 | 1 | 1 | 0 | 1 | 1 | 0 | 1 | 6 |
| Takamizawa,2020 | 1 | 1 | 1 | 0 | 1 | 1 | 1 | 1 | 7 |
| Xie,2020 | 1 | 1 | 1 | 0 | 1 | 1 | 1 | 1 | 7 |
| Akabane,2021 | 1 | 1 | 1 | 0 | 1 | 1 | 1 | 1 | 7 |
| Hiramatsu,2021 | 1 | 1 | 1 | 0 | 1 | 1 | 1 | 1 | 7 |
| Guc,2022 | 1 | 1 | 1 | 0 | 1 | 1 | 1 | 1 | 7 |
| Jin,2022 | 1 | 1 | 1 | 0 | 1 | 1 | 1 | 1 | 7 |
| Martínez-Escribano,2022 | 1 | 1 | 1 | 0 | 1 | 0 | 1 | 1 | 6 |
| Mazaki,2022 | 1 | 1 | 1 | 0 | 1 | 1 | 1 | 1 | 7 |
| Pian,2022 | 1 | 1 | 1 | 0 | 1 | 1 | 1 | 1 | 7 |
| Xie,2022 | 1 | 1 | 1 | 0 | 1 | 0 | 1 | 1 | 6 |
| Kim,2023 | 1 | 1 | 1 | 0 | 1 | 1 | 1 | 1 | 7 |
| Lu,2023 | 1 | 1 | 1 | 0 | 1 | 1 | 0 | 1 | 6 |
| Okamoto,2023 | 1 | 1 | 1 | 0 | 1 | 1 | 1 | 1 | 7 |
| Cozzani,2024 | 1 | 1 | 1 | 0 | 1 | 1 | 1 | 1 | 7 |

Table S3. Meta-regression analysis for the association of confounding factors and the hazard ratio for overall survival.

| **Covariates** | Overall survival | | |
| --- | --- | --- | --- |
|  | β value | P value | 95% CI |
| Publication year | -0.3374 | 0.2082 | -0.8629-0.1881 |
| Country | -0.0221 | 0.8660 | -0.2787-0.2345 |
| Sample size | -0.1834 | 0.5956 | -0.8606-0.4938 |
| Primary treatment | 0.0876 | 0.7516 | -0.4550-0.6302 |
| Reference value of CONUT score | 0.0124 | 0.9595 | -0.4662-0.4910 |
| TNM stage | -0.1672 | 0.2354 | -0.4433-0.1089 |
| Tumor location | 0.3752 | 0.1999 | -0.1985-0.9488 |
| Multivariate analysis | 0.1607 | 0.3649 | -0.1870-0.5085 |
| NOS | 0.0362 | 0.9155 | -0.6327-0.7051 |


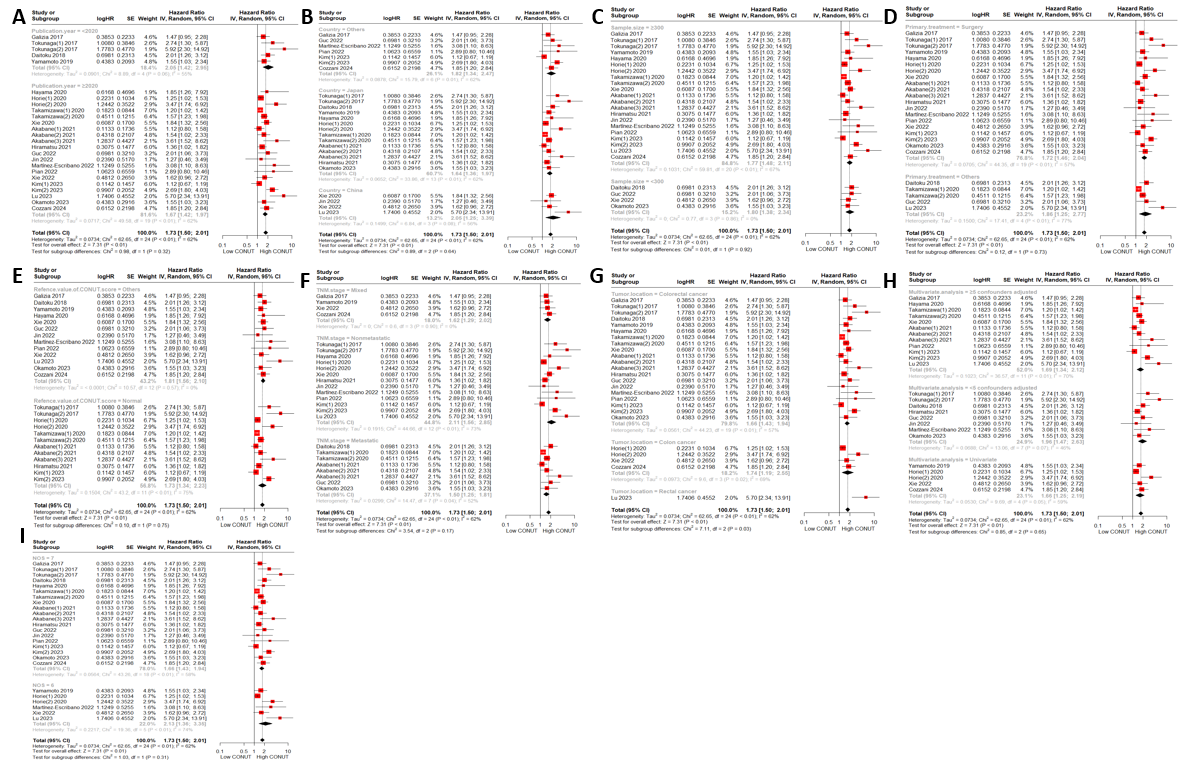


Figure S1. Forest plot of subgroup analyses assessing the relationship between the pretreatment CONUT score and overall survival. A: Publication year (<2020 vs. ≥2020); B: Country (China vs. Japan vs. Others); C: Sample size (<300 vs. ≥300); D: Primary treatment (Surgery vs. Others); E: Reference value of CONUT core (Normal vs. Others); F: TNM stage (Non-metastatic vs. Mixed vs. Metastatic); G: Tumor location (Colorectal cancer vs. Colon cancer vs. Rectal cancer); H: Multivariate analysis (≥5 common covariates adjusted vs. <5 common covariates adjusted vs. Univariate); I: NOS (6 vs. 7).


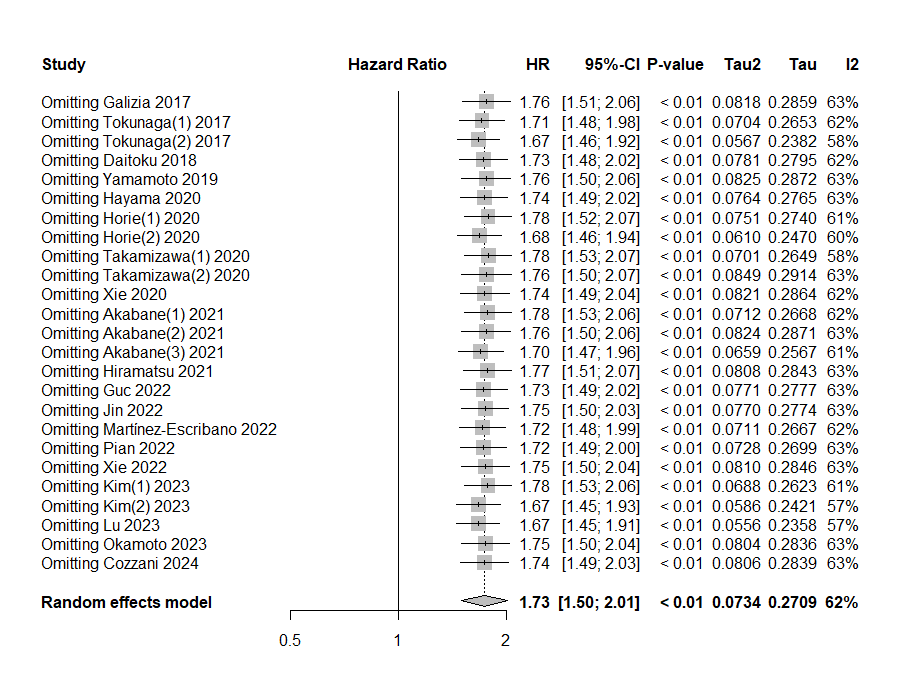


Figure S2. Sensitivity analyses assessing overall survival.
